# Knee Pain: The Fine Line Between Malignant and Benign

**DOI:** 10.7759/cureus.87790

**Published:** 2025-07-12

**Authors:** Andrew Lew, Suleman Janjua, Tariq M Awan, Nicholas Moore, Gregory M Cibor, Paolo Balmaseda, David M Peck, Michael P Montico

**Affiliations:** 1 Family Medicine, University of Nevada, Reno, Reno, USA; 2 Sports Medicine, Ascension Providence Hospital, Southfield, USA

**Keywords:** benign, chronic pain, edema, focal periphyseal edema, fope, knee, knee pain, salter-harris fracture

## Abstract

Focal periphyseal edema (FOPE) is a normal physiological finding that is often incidentally discovered on MRI of the knee. FOPE zones are areas of periphyseal edema typically observed near the time of physeal closure. This common physiologic phenomenon is related to changes in the distribution of forces around the physis as it closes during adolescence, occurring more frequently in females between 11 and 14 years of age. The condition may be associated with pain or may be asymptomatic. It is often mistaken for pathological bone marrow edema or a Salter-Harris fracture.

We present the case of a 14-year-old female patient with chronic knee pain and no history of trauma. MRI revealed two FOPE zones in the distal femur, with no other abnormalities. The patient was managed conservatively with observation and physical therapy, resulting in gradual symptom resolution. This case reinforces that FOPE should be recognized as a normal variant in adolescent knees to avoid misdiagnosis and unnecessary treatment.

## Introduction

First described in 2011, focal periphyseal edema (FOPE) of the knee refers to transient bone marrow edema centered around the proximal tibial physis and distal femoral physis, most commonly observed during the process of physiologic epiphyseal fusion [1,2]. It typically presents in adolescents aged 11-14, often in active individuals, and may or may not be associated with pain or a history of trauma [1]. Patients can be further classified based on likely MRI findings and the presence or absence of symptoms, distinguishing between symptomatic and incidental FOPE. FOPE can present in unilateral or bilateral knees, as well as in the hips [1,3,4].

MRI is the most sensitive modality for detecting FOPE, revealing focal hyperintense signals on fluid-sensitive sequences [5]. Given the nature of FOPE, findings on X-ray and CT are often equivocal. On MRI, FOPE may be misinterpreted as other pathologies with similar imaging features, including stress fractures, chronic osteomyelitis, and contusional bone marrow edema [1,6,7]. Therefore, it is essential to correlate imaging findings with a detailed history, including the mechanism of injury, physical examination, and clinical context. One important differential diagnosis is a Salter-Harris fracture, which involves the physis of long bones and requires timely treatment [8].

## Case presentation

Our case involves a 14-year-old female who presented to the hospital for evaluation of intermittent right anteromedial knee pain persisting for the past three months, first noticed while running during track practice. She has no significant medical history, recent illness, or relevant family medical history on file, and denies any injury or trauma. Her recent office visit revealed unremarkable clinical findings, and her X-rays were normal for her age.

**Figure 1:**
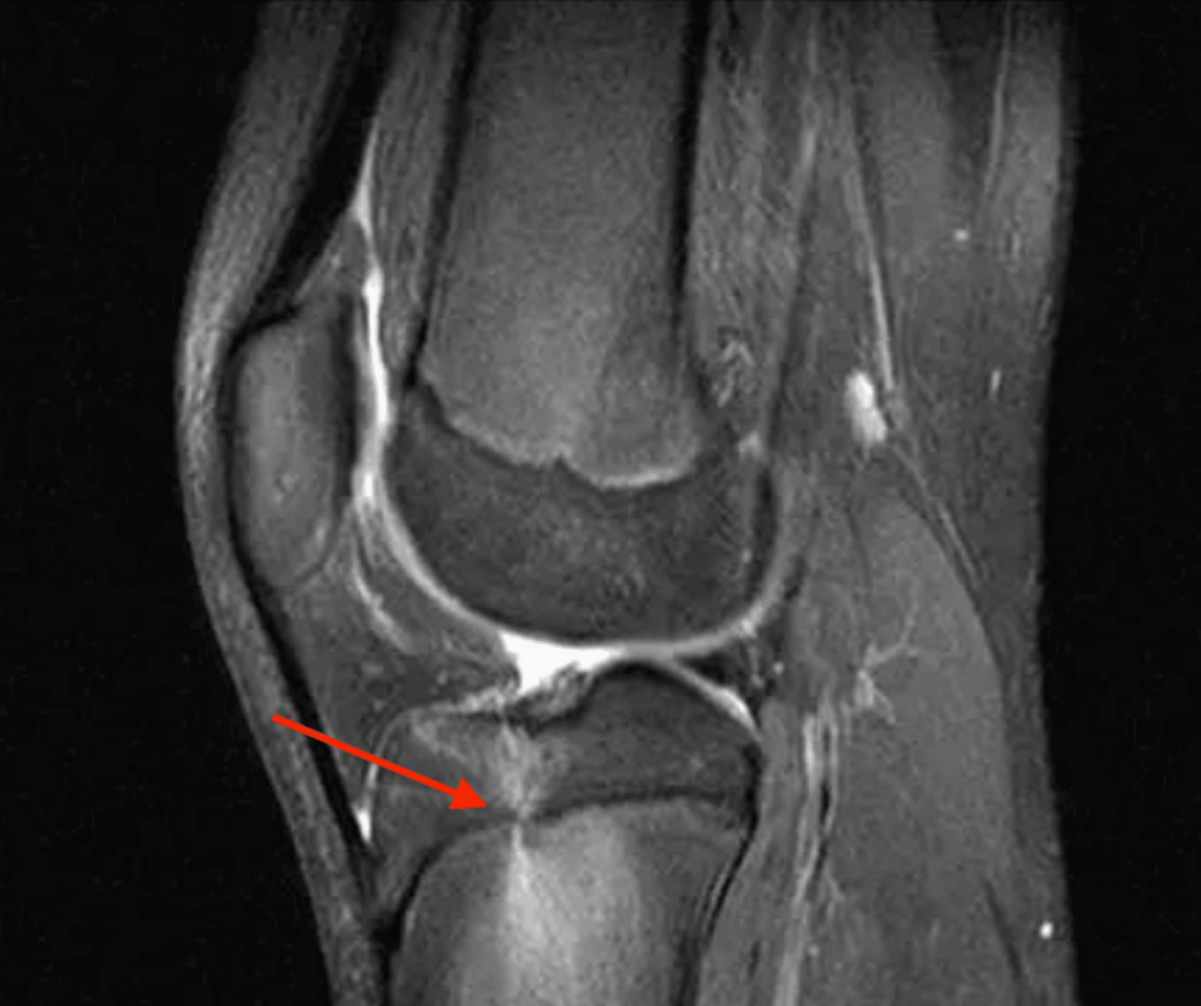
T2-weighted, fat-suppressed sagittal view of the knee showing hyperintensities in the proximal tibial physis (arrow).

**Figure 2:**
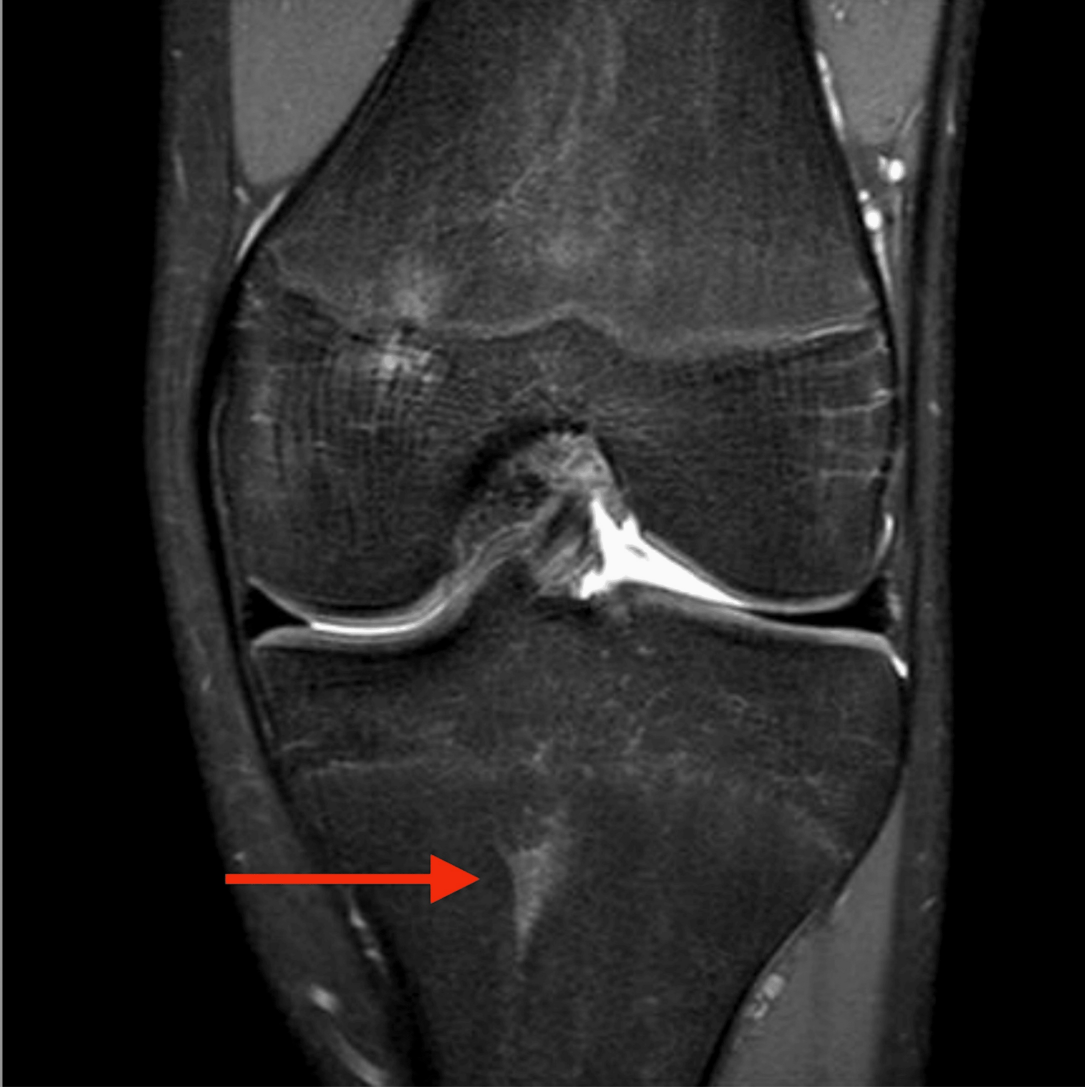
T2-weighted, fat-suppressed anteroposterior view of the knee showing hyperintense signals in the proximal tibial physis (arrow).

On physical examination, her right knee showed no erythema or effusion, and there were no signs of infection or ligament involvement. She exhibited mild tenderness over the anteromedial knee. She had a full range of motion with bilateral hyperextension (-5° to 130° of flexion), and motor and sensory function were intact distally. Initially, her symptoms raised clinical suspicion for patellofemoral syndrome, prompting an MRI. The MRI revealed two focal zones of hyperintense signals around the knee in the distal femur, consistent with early stages of physiologic physeal closure (Figures 1-2). These may be a source of pain, especially in the absence of any other abnormality or injury on MRI. She also followed up with orthopedic surgery and opted for conservative management, including rest, cryotherapy, and activity modification. She was referred to physical therapy for gentle stretching and strengthening through low-impact exercises. Her symptoms gradually improved over three months without the need for surgical intervention. Given the symptomatic improvement, no additional imaging studies were pursued during subsequent follow-up visits.

## Discussion

FOPE is a transient bone marrow edema pattern centered around the closing physis, most commonly seen in adolescents during the early stages of physeal fusion. It is often an incidental MRI finding and may or may not be associated with pain. Despite its appearance, FOPE is considered a benign and self-limiting process that does not require further diagnostic testing or treatment [1].

Three key studies were identified in the literature review exploring FOPE in adolescent patients. In a 2018 study titled “Focal periphyseal edema: Are we overtreating physiologic adolescent knee pain?”, Giles E et al. reported on four patients (three females, one male; average age 13.7 years) who presented with MRI findings consistent with FOPE. The majority were active in high-impact sports such as soccer, basketball, and running. Notably, none reported recent trauma or mechanical symptoms, and their physical exams were unremarkable, showing no swelling, atrophy, or range of motion limitations. Conservative management led to complete symptom resolution. MRI findings were consistent with FOPE [9].

Similarly, Zbojniewicz AM and Laor T (2011) proposed that FOPE represents a physiological process related to early physeal closure and may explain knee pain in adolescents when no other abnormalities are detected on imaging. They emphasized that the MRI appearance of FOPE, a bone marrow edema pattern centered on the physis, should not be misinterpreted as pathology. In their view, FOPE requires neither invasive diagnostics nor follow-up imaging [1].

Ueyama H et al. (2018) presented three FOPE cases with long-term clinical and imaging follow-up. Their findings supported the benign nature of FOPE, as none of the patients developed deformity or leg length discrepancy. They concluded that observation alone was sufficient, though further longitudinal studies may help validate this approach [10].

Our case aligns with these findings. The patient’s clinical improvement over time without invasive intervention makes FOPE the likely cause of her symptoms. This supports the prevailing theory that FOPE is a physiological response during physeal closure in adolescents and should be recognized as a normal variant to prevent misdiagnosis and overtreatment.

## Conclusions

As FOPE is a benign and self-limiting phenomenon that often coincides with normal physiologic physeal closure in adolescents, increased awareness among clinicians and radiologists can help distinguish it from more serious pathologies and guide appropriate care in the adolescent population.

As illustrated in our case, FOPE may present with nonspecific knee pain in the absence of trauma or other pathological findings. MRI is essential for accurate identification, and recognizing FOPE is crucial to avoid misdiagnosis and unnecessary interventions. Conservative management, including observation and physical therapy, is typically effective and remains the mainstay of treatment, with favorable outcomes. Despite its clinical relevance, FOPE remains a rare diagnosis, with only around 80 cases documented in the literature to date.
